# Reducing antimicrobial use in farm animals: how to support behavioral change of veterinarians and farmers

**DOI:** 10.1093/af/vfy006

**Published:** 2018-06-07

**Authors:** David C Speksnijder, Jaap A Wagenaar

**Affiliations:** 1 Department of Infectious Diseases and Immunology, Faculty of Veterinary Medicine, Utrecht University, Utrecht, The Netherlands; 2 University Farm Animal Practice, Harmelen, The Netherlands; 3 Wageningen Bioveterinary Research, Lelystad, The Netherlands

**Keywords:** antimicrobial resistance, prudent use, behavior change models

**Implications**

- Prudent use of antimicrobials in livestock requires the adoption of management and treatment practices which replace or reduce the need for antimicrobials by veterinarians and farmer.
- The adoption of these new practices equals behavior changes of these actors.
- Changing human behavior can be very difficult, especially when the behaviors of interest are routine behaviors.
- The use of social sciences can be of great value in understanding why veterinarians and farmers act in certain ways and how they can be motivated to change antimicrobial use practices.

## Introduction

To counteract the globally increasing threat of antimicrobial resistance, antimicrobials should be used prudently, both in humans and in animals. A general principle of prudent antimicrobial use in veterinary medicine is that animals receive antimicrobials appropriate to their clinical needs, and while the efficacy of these antimicrobials for treating animals and humans is sustained (Weese et al., 2013; OIE World Organization for Animal Health, 2016). This requires that prophylactic and growth promoting antimicrobial use practices should be abandoned and antimicrobial treatments should be diminished by minimizing the incidence of infectious diseases. When antimicrobial treatments are used to treat a bacterial infection, they should be carefully selected and administered based on prudent use principles (Weese et al., 2013; Aarestrup, 2015; Speksnijder et al., 2015a, Speksnijder et al., 2015b and Speksnijder et al., 2015c). The recently published WHO-Guideline aligns with these principles (Aidara-Kane et al., 2018) and suggest the need for measures that are partly dependent on the development and enforcement of regulations (e.g., stop the use of growth promotors). When it comes to on-farm decisions for treatment, behavioral aspects are as important as technical ones. Aspects like prior experience and risk avoidance have a major influence on the decision about treatment. Typically, farmers and veterinarians are responsible for implementing disease preventive measures and for prescribing and/or administering antimicrobials. There is extensive knowledge available to substantially prevent, reduce, and control the burden of many animal diseases without the use of antimicrobials in production animals; the battleground is in consistently and effectively implementing the necessary management changes (LeBlanc et al., 2006; Ruston et al., 2016). The adoption of prudent antimicrobial use principles thus requires a behavioral change in veterinarians and farmers. It is widely believed that the role of veterinarians should change from reactive and curative antimicrobial prescribers toward a more proactive role as animal health consultants for farmers, without relying on prescribing antimicrobials. Farmers should ideally depart from relying on using antimicrobials as a management tool toward a more proactive approach that prevents animal diseases, and uses antimicrobials only as a last resort (i.e., when preventive measures have failed). In practice, however, these behavioral changes seem rather difficult to accomplish (Garforth, 2015; Speksnijder et al., 2015a, b). The reasons why behavioral changes are often difficult to initiate and sustain in practice are complex and typically differ from person to person. The use of sociopsychological models might help to better understand the different factors that influence current behaviors and how to invoke behavioral changes in farmers and veterinarians related to antimicrobial use practices and the uptake of preventive measures. Insights into enabling or inhibiting factors can be helpful for policy makers, advisers, and others, which will ultimately help support veterinarians and farmers in adopting new behaviors (Kristensen and Jakobsen, 2011; Garforth, 2015).

## Human Behavior

**Figure 1.**
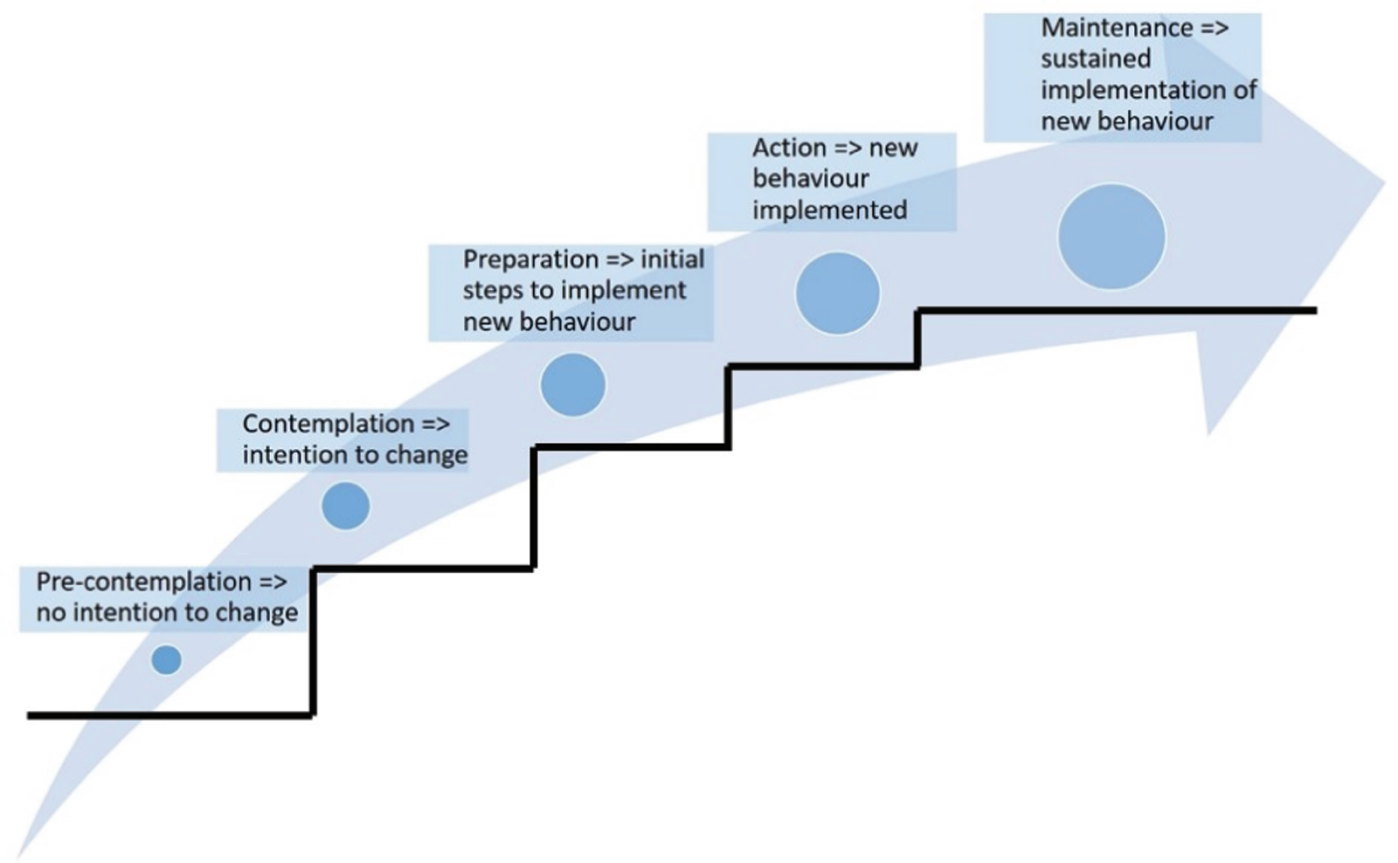
The transtheoretical model explained. This theory assumes that a change of behavior proceeds through several steps. From a precontemplation stage, during which an individual has no intention to change, to a contemplation stage, during which intention is developed through preparation and action stages, to a sustained change of behavior. To successfully induce behavioral change, individuals should receive support which is tailored to the stage of change they are in. In the early stages, this requires support to develop an intention to change. In the latter stages, this requires the presence of reinforcing factors to enable and stimulate sustained action. Derived from Prochaska (2013).

A number of sociopsychological models exist that offer insight into the complexities underlying human behavior and behavioral change. Increasingly, these models are being used in agricultural research (Edwards-Jones, 2006; Garforth, 2015; Jones et al., 2015; Coyne et al., 2016; Visschers et al., 2016), explaining human behavior using a set of intrinsic motivational factors and extrinsic enabling or inhibiting stimuli for performing this behavior. The Transtheoretical Model of Change developed by Prochaska (2013) assumes that, in order for an individual to change behavior, a person first should be aware of the need to change behavior and develop an intention to change. Subsequently, this intention to change needs to transition into action, followed by a maintenance phase where relapse into old behaviors should be prevented (Figure 1).

**Figure 2.**
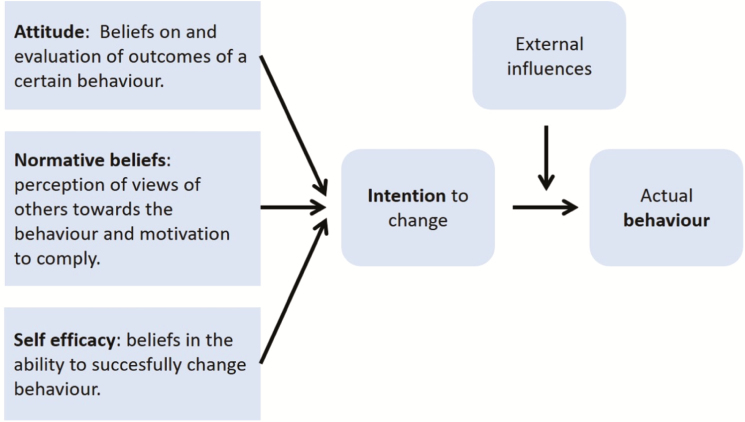
Theory of planned behavior explained. According to this model, an intention to change a behavior is influenced by beliefs regarding the outcome of a behavior, beliefs about perceptions of others toward the behavior and the beliefs in one’s own abilities to successfully change a certain behavior. Intention to change is often a good predictor of actual behavior under external influences (Ajzen, 2002).

A widely used model in social sciences to explain voluntary behavior change (which connects to the first three stages of the Transtheoretical model) is the theory of planned behavior (**TPB**; Ajzen, 1991). TPB assumes that the strength of an intention, also called the intrinsic motivation, to engage in a certain behavior is a predictor of actual behavior. The TPB is composed of three belief variables which together act as drivers or inhibitors for an intention to change. These beliefs are related to attitudes, perceived norms of others, and self-efficacy (Figure 2). Understanding people’s beliefs about a specific behavior can be useful in designing specific strategies to support people to adopt new behaviors.

Once an intention for a certain behavior is established, it needs to progress into action and should be sustained over time to have a lasting impact. Here, external factors come into play—factors which an individual often has limited control over. A person’s “hardware” (skills and knowledge) and environment can facilitate or restrict the performance of an actual behavior through resources, tools, education, subsidies, regulations, organizational constraints, fines, etcetera (Ellis-Iversen et al., 2010).

## Explaining Behaviors of Farmers and Veterinarians Related to Antimicrobial Use

Over the last decade, several studies have tried to understand and influence the beliefs and behaviors of farmers and veterinarians related to the prudent use of antimicrobials. The main findings of these studies are discussed below (Figure 3).

**Figure:**
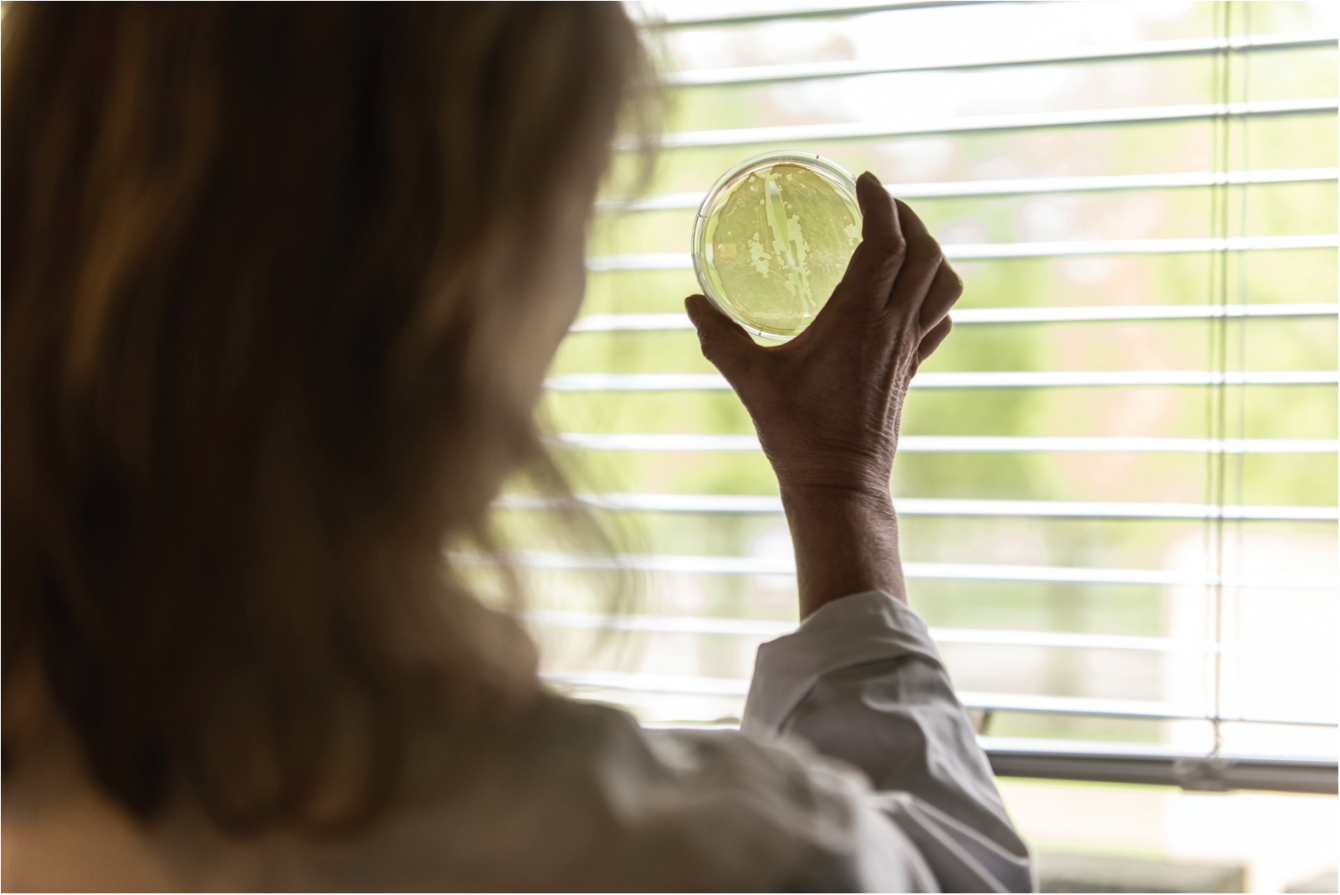


### Attitudes

Veterinarians and farmers do not always seem to be aware of the risks for public health related to the (extensive) use of antimicrobials in animals, and as such, do not always feel responsible for the problematic outcomes, which lowers their motivation to change (Speksnijder et al., 2015a; Coyne et al., 2016; Visschers et al., 2016; Ritter et al., 2017). In addition, antimicrobials seem to be often prescribed and/or applied by veterinarians and farmers as a risk avoiding strategy to prevent potential complications due to infectious diseases (Coyne et al., 2014; Speksnijder et al., 2015a, b). Although veterinarians increasingly advise farmers on specific management measures aimed at preventing animal diseases and reducing antimicrobial use, uncertainty regarding the (cost) effectiveness of these measures often hampers the implementation of these recommendations. Veterinarians regularly seem unable to clearly calculate the costs and/or efforts and benefits related to these measures (Speksnijder et al., 2015a, b, c; Ritter et al., 2017). Also, conflicting recommendations from different farm advisors (including veterinarians) can be a major obstacle for implementing veterinary advice (Speksnijder et al., 2015a; Ruston et al., 2016). Personal (bad or good) experience with specific antimicrobial use practices or management changes has been found to greatly influence the attitudes of farmers and veterinarians—especially when exploring new management or antimicrobial use routines (Alarcon et al., 2014; Alarcon et al., 2014; Speksnijder et al., 2015a; Ritter et al., 2017)

### Normative Beliefs

Veterinarians are widely seen by farmers as the major referent for animal health and as a result, a farmer’s intention to reduce antimicrobial use and change management practices based on influence from a veterinarian has been observed (Gunn et al., 2008; Jones et al., 2015; Visschers et al., 2016). However, veterinarians have been found to often fall short in adopting this supportive role as proactive and motivating animal health consultants (Gunn et al., 2008; Alarcon et al., 2014; Laanen et al., 2014; Jones et al., 2015). Perceived social pressure and “social norms” within a farmer community also have a great influence on a farmer’s intentions to engage in different behaviors related to disease control and antimicrobial use practices (Ellis-Iversen et al., 2010; Alarcon et al., 2014; Coyne et al., 2014; Ritter et al., 2017).

Veterinarians often see their main role as a service provider to farmers, who are perceived to want fast and cheap solutions to animal health problems. Studies have shown that veterinarians strongly sympathize with both perceived negative attitudes toward disease control measures (e.g., questioning the efficacy of disease control measures propagated by scientists, extension services, and others) and the financial constraints faced by farmers in implementing preventive measures. Inasmuch, they often sense a lack of demand from their clients for advice (Gunn et al., 2008; Alarcon et al., 2014; Coyne et al., 2016). The fear of losing a client after a wrong therapy decision and/or for being intrusive when advising management changes, as well as a perceived demand for prescriptions by farmers, might greatly influence the daily practices of veterinarians (Speksnijder et al., 2015a; Coyne et al., 2016). On the other hand, veterinarians often observe a societal urge to reduce antimicrobial use, which might result in a complex web of interests. Support from colleagues and veterinary guidelines might help veterinarians to take more responsibility regarding the prudent use of antimicrobials—regardless of the (perceived) wishes and demands of farmers—in order to better address public and animal health.

### Beliefs in Abilities; Self-Efficacy

Veterinarians, in general, are believed to have proper technical knowledge to advise on measures which can substantially decrease the burden of animal diseases and antimicrobial use at the farm level. However, veterinarians often face difficulties putting this knowledge into practice and motivating farmers to implement recommended changes (Speksnijder et al., 2015b). Research has shown that veterinarians can feel insecure about their own advisory and communication skills, perceive insufficient support from colleagues in their advisory role, and feel insecure to act as advisor among all kinds of other farm advisors (Gunn et al., 2008; Ruston et al., 2016). Veterinarians often perceive it beyond their control (and probably also beyond their responsibility) to impose changes in farmers’ behaviors, and may therefore, acquiesce in the status quo when they believe that their farmers cannot be motivated to change their management (Speksnijder et al., 2015b). It has been proven that the more farmers and veterinarians believe in their abilities, and experience positive outcomes in controlling animal diseases with less or no use of antimicrobials, the higher their intention becomes to actively engage in practices that reduce antimicrobial use (Coyne et al., 2014; Jones et al., 2015; Visschers et al., 2016). However, substantial proportions of farmers and veterinarians see few feasible alternatives to the use of antimicrobials to control animal diseases in the current husbandry systems and do not believe that they can effectively operate using less antimicrobials (Kristensen and Jakobsen, 2011; Coyne et al., 2014; Visschers et al., 2016). This will obviously negatively influence the motivation of both farmers and veterinarians to engage in activities aiming to reduce antimicrobial use. When farmers lack belief in their peers to successfully engage in disease preventive measures, they might also lose confidence in their own beliefs around effectively controlling diseases (Gunn et al., 2008; Ellis-Iversen et al., 2010). The perception of farmers toward the feasibility (costs, labor, possibilities within the physical make-up of the farm) also greatly influences farmers’ intentions to implement specific management measures (Gunn et al., 2008; Alarcon et al., 2014; Speksnijder et al., 2015b; Ritter et al., 2017).

### From Motivation to Actual Behavior: External Influences

Once an intention to change is present, personal abilities and external factors should stimulate and enable the implementation of disease preventive measures and changes in antimicrobial use practices. Governments can introduce coercive instruments, such as regulations and fines, to induce behavior changes, but these might also introduce unforeseen and unwanted side effects (e.g., illegal use practices, animal welfare issues) and require significant capacity from inspection authorities. They can also support veterinarians and farmers to engage in voluntary behavior changes by means of provisions, education, and social pressure (Speksnijder et al., 2015c). The Dutch approach in the last decade has shown that a combination of policy—setting strict reduction targets for antimicrobial use—and supportive instruments can have a huge effect on the level of antimicrobial use in farm animals (Speksnijder et al., 2015c). Benchmarking of antimicrobial prescribing and use might enable farmers and veterinarians to calibrate their frame of reference compared with peers. In both Denmark and the Netherlands, this has shown to be very effective and might open discussions between farmers and veterinarians around the level of antimicrobial use and specific antimicrobial use practices (Jensen et al., 2014; Speksnijder et al., 2015b). Public pressure has been a major driver in several countries to reduce the use of antimicrobials in farm animals. It can serve as an accelerator for further action, as was clearly the case in the Netherlands (Speksnijder et al., 2015b).

Some factors influencing disease dynamics and antimicrobial use are beyond the control of individual farmers and veterinarians. These external factors (for example, the state of immunity of animals arriving at a farm, feed quality) ultimately limit a farmer’s or veterinarian’s operating framework. The presence of such external factors demands good collaboration throughout the whole production chain (Speksnijder et al., 2015a)—especially among farmers, veterinarians, and nonveterinary advisers. Many studies have pointed out that management recommendations from different advisers often lack harmony, and sometimes even completely conflict. Nevertheless, farmers and stakeholders consistently promote the added value that comes of continuous support from different advisers regarding management measures, further stressing the need for consistent information and communication around the cost and/or benefits of reducing antimicrobial use (Sayers et al., 2014; Ruston et al., 2016; Speksnijder et al., 2017).

Proper stockmanship skills are essential in taking prompt management actions to curb the risk of animal diseases; however, not all farmers possess these skills (Ellis-Iversen et al., 2010; Speksnijder et al., 2015a). Veterinarians and other advisers may try to coach and/or mentor such farmers in the right direction. However, at the end of the day, veterinarians are dependent on farmers’ degree of compliance in implementing preventive measures. This compliance is also related to the available resources of a farmer (finances, labor) and physical characteristics of the farm (buildings, location, etc.), which may restrict the implementation of certain measures to reduce antimicrobial use (Speksnijder et al., 2015a). A huge challenge for veterinarians is in translating technical veterinary knowledge into practical advice which is tailored to the unique situation of the farmer. Many studies have pointed out that veterinarians often fall short in this area, and too often act as a preacher rather than a psychologist, whose aim is to elicit a farmers’ true goals, needs, beliefs, and risk perceptions (Kristensen and Jakobsen, 2011; Sayers et al., 2014; Ruston et al., 2016; Ritter et al., 2017). Changing this dynamic is incredibly important, but undoubtedly poses a huge challenge for farm animal veterinarians moving forward.

## Putting it in Practice

Some major recommendations to promote the prudent use of antimicrobials can be distilled from the findings above. There is a clear need to communicate the need for restrictive use of antimicrobials through information channels that are perceived trustworthy by farmers (Ritter et al., 2017). Veterinarians are generally perceived as trustworthy referents for farmers and, therefore, veterinarians should act as the main information source on prudent use of antimicrobials to farmers. For veterinarians, the professional veterinary associations, scientific and professional veterinary journals, and peer meetings are often regarded as trusted information sources, and they could potentially serve as effective information channel to reach veterinarians. The professional veterinary associations, in collaboration with governments and other important stakeholders, should make clear that a cogent and uniform message is communicated. Also, best practices to reduce antimicrobial use in a sustainable way should be communicated via these information channels to inform veterinarians and farmers and encourage them to copy these best practices (Ellis-Iversen et al., 2010). To withstand (perceived) pressure from farmers, professional standards and guidelines may have their value in harmonizing the prescribing behavior of veterinarians and enable veterinarians to act as independent professionals (Speksnijder et al., 2015b).

For governments, it might be advisable to stimulate and enable front running initiatives which aim to lower antimicrobial use in food-producing animals. Several policy options are available for this purpose, ranging from provisions, education, and investments in research on the development of best management practices. However, exclusively voluntary approaches to control antimicrobial use in food-producing animals have hardly ever been effective. A comprehensive mix of coercive and voluntary approaches seems to be the most effective approach in effectuating reductions in antimicrobial use at a country level.

A crucial aspect of herd health management and reducing the need for antimicrobials is the harmonization of advice of different advisors, as this will lead to greater trust with farmers and greater reception to the provided information (Sayers et al., 2014). Ample evidence is available that farmers’ intention to change is greatly reinforced by mutual support from their major referents: veterinarians and other advisers (Ellis-Iversen et al., 2010). If veterinarians want to make the shift from curative work to a preventive advisory role, they should invest in coaching and/or mentoring skills, such as trust, empathy, listening skills, tact and diplomacy, competency, dedication, honesty, and openness, in order to guide farmers in the right direction (Kristensen and Jakobsen, 2011). Veterinarians should be able to understand which stage of change a farmer is in (Figure 1) and adapt their message accordingly: they should try to create awareness and persuade farmers with no intention to change, educate farmers on how to change when they have gathered an intention, and reinforce farmers who have started to make a change. It is very important that veterinary advisory activities offer farmers a sense of ownership over the recommendations. Rather than of simply putting advice on the desk of a farmer, it is crucial to mutually discuss recommendations with them—for evidence has shown that farmers prefer and appreciate this tact, and as a result, are far more likely to implement recommendations offered through this approach (Hall and Wapenaar, 2012; Speksnijder et al., 2017).

## Conclusion

Changing human behavior is complex. However, insights into the drivers and barriers for behavior change will help develop interventions to support behavior change. The use of sociopsychological models can be of great value in curbing the emerging threat of antimicrobial resistance arising from indiscriminate use of antimicrobials in food animal production. These insights can be used by policy makers to develop effective policies aimed at a reducing antimicrobial use. They can also be used by veterinarians in their advisory role at the farm level to influence the management of individual farmers. This will ultimately improve animal health and lower the need for antimicrobial use.

## Remark

This manuscript is an adapted and shortened version of the general discussion of the Ph.D. thesis of Dr. David Speksnijder published in 2017: Antibiotic use in farm animals; supporting behavioral change of veterinarians and farmers. These results have been presented at the RuVASA congress, 31 May to 2 June 2017, Pretoria, South Africa.

## Funding

The PhD study was funded by the Netherlands Organization for Health Research and Development (ZonMW); grant number 205100009.

## About the Authors

**Dr. D.C. (David) Speksnijder**, DVM, Ph.D, works as researcher in the field of infectious diseases and antimicrobial use and resistance in farm animals in the Department of Infectious Diseases and Immunology, Faculty of Veterinary Medicine at Utrecht University. He is currently working in the University Farm Animal Practice. His research focuses on reducing antimicrobial use through behavioral changes of farmers and veterinarians, both in the national and the international context.

**Prof. J.A. (Jaap) Wagenaar**, DVM, Ph.D., is currently working as a professor in the Department of Infectious Diseases and Immunology, Faculty of Veterinary Medicine at Utrecht University. His research area covers the transmission of bacteria between animals and human, and the emergence of antimicrobial resistance—in particular ways to intervene in the increasing problem of resistance. Prof. Wagenaar is member of several national and international working groups on antimicrobial resistance and acts frequently as advisor for WHO, FAO, and OIE.
